# An Overview of the Polymorphisms of Circadian Genes Associated With Endocrine Cancer

**DOI:** 10.3389/fendo.2019.00104

**Published:** 2019-02-26

**Authors:** Sonia Morales-Santana, Santiago Morell, Josefa Leon, Angel Carazo-Gallego, Jose C. Jimenez-Lopez, María Morell

**Affiliations:** ^1^Proteomic Research Service, San Cecilio University Hospital, Instituto de Investigación Biosanitaria de Granada (Ibs.GRANADA), Granada, Spain; ^2^Department of Genetics, University of Cambridge, Cambridge, United Kingdom; ^3^Clinical Management Unit of Digestive Disease, San Cecilio University Hospital, Instituto de Investigación Biosanitaria de Granada (Ibs.GRANADA), Granada, Spain; ^4^Genomic Research Service, San Cecilio University Hospital, Instituto de Investigación Biosanitaria de Granada (Ibs.GRANADA), Granada, Spain; ^5^Department of Biochemistry, Cell and Molecular Biology of Plants, Estación Experimental del Zaidín, Spanish National Research Council (CSIC), Granada, Spain; ^6^The UWA Institute of Agriculture and School of Agriculture and Environment, The University of Western Australia, Perth, WA, Australia; ^7^Genomic Medicine Department, GENYO, Centre for Genomics and Oncological Research, Pfizer/University of Granada, Andalusian Regional Government, PTS Granada, Granada, Spain

**Keywords:** polymorphism, circadian clock genes, endocrine cancer, breast cancer, ovarian cancer, prostate cancer, pancreatic cancer, thyroid cancer

## Abstract

A major consequence of the world industrialized lifestyle is the increasing period of unnatural light in environments during the day and artificial lighting at night. This major change disrupts endogenous homeostasis with external circadian cues, which has been associated to higher risk of diseases affecting human health, mainly cancer among others. Circadian disruption promotes tumor development and accelerate its fast progression. The dysregulation mechanisms of circadian genes is greatly affected by the genetic variability of these genes. To date, several core circadian genes, also called circadian clock genes, have been identified, comprising the following: *ARNTL, CLOCK, CRY1, CRY2, CSNK1E, NPAS2, NR1D1, NR1D2, PER1, PER2, PER3, RORA*, and *TIMELESS*. The polymorphic variants of these circadian genes might contribute to an individual's risk to cancer. In this short review, we focused on clock circadian clock-related genes, major contributors of the susceptibility to endocrine-dependent cancers through affecting circadian clock, most likely affecting hormonal regulation. We examined polymorphisms affecting breast, prostate and ovarian carcinogenesis, in addition to pancreatic and thyroid cancer. Further study of the genetic composition in circadian clock-controlled tumors will be of great importance by establishing the foundation to discover novel genetic biomarkers for cancer prevention, prognosis and target therapies.

## Introduction

Circadian clocks have been defined as endogenous oscillators that synchronize daily both physiological and behavioral rhythms with local time cues (1, 2). In mammals, this evolutionary adaptation provides a survival advantage to anticipate environmental changes and allowing them to modify their daily rhythms in the most efficient way to meet these environmental changes (1, 3, 4).

Consequences of modern lifestyle as sleep deprivation, shift work time schedule, altered mealtime or excessive artificial light exposure at night promote metabolic imbalances that can alter the circadian system. Interestingly, dysfunction of the molecular clock is linked to uncontrolled cell proliferation in human cancer (5–7). Changes in circadian clock increase risk of different cancers in endocrine tissues which require daily proliferation to carry out their activity. Thus, a considerable part of endocrine signals are clock-controlled (8). The dysregulation mechanisms of the circadian clock genes is greatly affected by polymorphic variants of circadian clock-related genes, which can raise human cancer risk through different physiological systems (9).

This mini-review focused on the role of single polymorphisms of circadian clock genes that similarly to environmental factors might increase the risk of developing human endocrine cancers.

## Circadian Clock Mechanism, Clock Genes, and Cancer

The circadian clock regulates both physiology and behavior according to the daily cycle of light and dark. In mammals, it is hierarchically organized and integrates the master clock, which is located in the suprachiasmatic nucleus (SCN) within the hypothalamus, and the peripheral clocks as well, ubiquitously found virtually in all peripheral tissues and cells (10). SCN clock is constantly coupled to environmental cues, mainly photoperiod, through the photic signals from the retina (11), daily rhythms in temperature, diet and social phenomena through a complex downstream neurohumoral pathways. Oscillators located in brain nuclei and peripheral tissues are also connected by SCN clock (12).

Circadian rhythms are controlled by circadian pathway genes. The molecular circadian clock is originated by a transcriptional/translational loop of circadian clock genes with autoregulatory feedback. The primary loop involves the genes *CLOCK, BMAL1* (also known as *ARNTL1), PER1–3*, and *CRY1-2*. During the day, the complex integrated by CLOCK and BMAL1 stimulates the expression of negative regulators period genes (*PER1–3*) and cryptochrome genes (*CRY1-2*). Heterodimers constituted by PER and CRY operate as co-repressors, binding to the CLOCK-BMAL1 complex and inhibiting *CRY* and *PER* gene transcription induced by CLOCK-BMAL1. Furthermore, in the dark phase, *CRY* and *PER* expression decrease to the CRY-PER repressor complex. This leads to a new cycle of the transcription activation of the *CLOCK-BMAL1* complex, which completes the basic auto regulatory loop (8, 13). Otherwise, different modulators display fine tuning of output signals in molecular clock.

Currently, several core circadian genes, also known as circadian clock genes, have been identified in humans (13): *ARNTL* (aryl hydrocarbon receptor nuclear translocator like, also identify in brain and muscle as Arnt-like protein-1, BMAL1) (14, 15), *CLOCK* (clock circadian regulator) (16), *CRY1* (cryptochrome circadian clock 1), *CRY2* (cryptochrome circadian clock 2) (17), *PER1* (period circadian clock 1), *PER2* (period circadian clock 2), *PER3* (period circadian clock 3) (18–20), *CSNK1E* (casein kinase I epsilon) (6, 21), *NPAS2* (neuronal PAS domain protein 2) (22, 23), *NR1D1* (nuclear receptor subfamily 1 group D member 1 also called Rev-Erb alpha) (24, 25), *NR1D2* (nuclear receptor subfamily 1 group D member 2 also referred to Rev-Erb beta) (26), *RORA* (RAR related orphan receptor A) (27) and *TIMELESS* (timeless circadian clock) (28, 29).

In addition, there is a large amount of circadian genes from other clock-related pathways.

Ground-breaking works in the field of molecular cancer epidemiology have unveiled a large quantity of polymorphisms affecting the clock genes (Table 1). Studies on the relationship between clock genes polymorphisms and cancer susceptibility have established that variants of *ARNTL, CLOCK, CK1*ε*, CRY1-2, NPAS2*, and *PER1-3* are frequently associated to human reproductive tissues and pancreatic cancers (1).

**Table 1:** Genetic association between circadian clock polymorphisms and different endocrine-related cancers.

**Circadian gene**	**dbSNP ID**	**Cancer types**	**Phenotype**	**References**
ARNTL	rs3816358	Breast	Reduced risk	(30)
	rs7950226	Prostate	Increased risk	(31)
	rs142435152		Risk associated	(4)
	rs10732458	Ovarian	Increased risk	(32)
	rs117104877		Decreased risk	
CLOCK	rs11133373	Prostate	Decreased risk	(31)
	rs3805151	Breast	Increased risk	(33)
	rs10462028		Increased risk	(30)
CRY1	rs12315175	Prostate	Increased risk	(34)
	rs7297614 and rs1921126		Fatal disease	(35)
	rs10778534		Increased risk	(36)
	rs1056560	Breast	Heterozygous variant genotype decreased risk	(33)
CRY2	rs1401417	Breast	Decreased risk	(33)
	rs1401417	Prostate	Increased risk	(37)
	rs2292912		Decreased risk	(31)
CSNK1E	rs135750	Ovarian	Increased risk	(32)
	rs1534891	Prostate	Increased risk	(31)
NPAS2	rs2305160	Prostate	Decreased risk (lower insulin resistance)	(37)
	rs895521 and rs1369481		Decreased risk	(31)
	rs17024926		Increased risk	
	rs23051560	Breast	increased risk	(38)
PER1	rs885747		Heterozygous variant genotype decreased risk	(31)
	rs2289591		Heterozygous variant genotype increased risk	
PER2	rs7602358	Prostate	Increased risk	(31)
	rs934945	Breast	Increased risk together with CLOCK homozygous variant genotype	(33)
PER3	rs1012477	Prostate	Increased risk	(31)
	rs228697		Decreased risk	(36)
RORA	rs17191414	Prostate and breast	Risk associated	(4)
	rs1482057 and rs12914272	Breast	Risk associated	(39)
	rs12913421	Pancreatic	Decreased risk	(40)
TIMELESS	rs2291738	Breast	Decreased risk	(41)
	rs7302060	Breast	Decreased risk	

## Breast Cancer

Epidemiological studies have concluded an association between shift work and breast cancer risk (42–44). However, currently available experimental/epidemiological data are characterized by a great heterogeneity and some findings are in disagreement with previous observations about relationship between shift work and breast cancer risk (45).

A recent study in breast cancer showed that circadian genes CRY2 and *P*ER1-3 were down-regulated, while *CLOCK* and *TIMELESS* were over-expressed (46). This study confirms previous results where *PER1, PER2*, and *PER3* exhibited changes associated with the tumor suppressor activity (47, 48). In breast epithelial cells, an altered estrogen receptor signaling has been related to breast cancer and two circadian clock genes, *PER2* and *ARNTL*, both required for breast epithelial acinar morphogenesis *in vitro* (49).

Therefore, polymorphism studies in circadian clock-related genes and breast cancer, most of them have focused on the evaluation of the polymorphisms in core circadian genes, melatonin biosynthesis and signaling pathways. In this line, polymorphisms affecting *CLOCK* (rs3805151), *CRY1* (rs1056560), *CRY2* (rs1401417), and *PER2* (rs934945) (33) have been associated to breast cancer risk in a Chinese population. Concerning *CLOCK* gene, it was found that carrier subjects CT and CT+TT genotypes exhibited an increased risk of breast cancer in comparison to CC carriers. However, the GT genotype of the *CRY1* gene was the genetic variant with the lower risk of suffering cancer, as well as for those subjects carrying CC genotype of the *CRY2* gene having an even lower risk. Finally, simultaneous presence of *CLOCK* CC and *PER2* AA genotypes resulted in a higher risk of developing breast cancer (33).

An alternative study supporting the involvement of the *CLOCK* gene in the development of breast cancer came from a Bonn (Germany) population of shift workers. This case-control study analyzed the associations between breast cancer and polymorphisms in circadian clock genes (*ARTNL, CLOCK, CRY2, NPAS*, and *PER2*) and in genes of the melatonin pathway as well (*AANAT* and *MTNR1B*). In these workers with the rs8150 polymorphism from *AANAT* gene, as well as for the rs10462028 polymorphism from the CLOCK gene, was observed an increased frequency of breast cancer, while a lower risk was observed for the rs3816358 from ARNTL gene (30). Interaction analysis was performed in two-ways, detecting interactions between shift work and *CLOCK* gene. Therefore, interactions were found for shift work and *MTNR1B, ARNTL*, and *NPAS2* genes (50).

Additionally, a combined analysis of three genome wide associations studies (GWAS) was performed, including a total 285,984 SNPs (Single Nucleotid Polymorphisms) in an European population of 2.702 women with invasive breast cancer, in comparison to 5,726 subject controls. The most important association to breast cancer was found for genes implicated in the circadian clock (51). Monsees et al. conducted a study on 1,825 women finding common variants across 15 circadian system genes that were tested for association with breast cancer risk. The interaction study was designed in a subset of 1,318 women, showing interactions between genotype and rotating shift-work. *NPAS2* gene was strongly associated to the risk of developing breast cancer, and the rs23051560 variant was related with potential effects. The estimated risk value for the minor allele (A) was lower among women with <2 years of shift-work compared to women with more than 2 years of shift-work (38).

In Europe, among a French population analyzed, 577 SNPs were found in 23 circadian clock genes associated with breast cancer risk in more than 1,000 cases and control subjects. *RORA* SNPs rs1482057 and rs12914272148 were also associated with breast cancer (39). Furthermore, a significant association has been shown between two SNPs in the *TIMELESS* gene (rs2291738 and rs7302060) (33, 41). In this study, C allele and CC genotype from rs7302060 polymorphism were associated with lower breast cancer risk. In rs2291738 polymorphism, GG genotype was related to decreased breast cancer risk.

On the other hand, limitations of the selected studies include the stratification of the populations according to different conditions among the different studies, such as shift work duration, menopausal status and expression levels of hormonal receptor in tumor tissues. Moreover, the polymorphism associations to breast cancer risk remain controversial, since some of these results were not able to be replicated in other populations or significance of associations is lost after adjusting for confounding factors. Furthermore, the described investigations included other limitations which will be further discussed in more detail.

## Prostate Cancer

Prostate cancer exhibits the highest cancer prevalence in men, being the second cause of cancer-related deaths (52). Normal prostate cancer development is dependent on androgens levels. Circadian clock genes regulate androgen production (53), affecting prostate cancer evolution (54). On the other hand, a balanced regulation of the circadian clock genes might modulate and even suppress tumor growth by controlling DNA replication, repair mechanisms and cell proliferation (55). Although a limited number of epidemiologic studies have been realized, several circadian genes have been implicated in prostate cancer regulation: *ARNTL, CLOCK, CRY1-2, CSNK1e, MTNR1A* and *MTNR1B, NPAS2, NR1D1, PER1*-3, *RORA, RORB*, and *TIMELESS* (4, 31, 56, 57).

One of the first epidemiologic studies performed on prostate cancer and their associated SNPs, Chu et al. identified five polymorphisms in five circadian genes. This case-control study was conducted in a Chinese population with 187 cases and 242 control subjects (37). These polymorphisms encompassed *CRY2* rs1401417, *CSNK1E* rs1005473, *NPAS2* rs2305160, and *PER1* rs2585405. The C allele from *CRY2* presented an elevated prostate cancer risk compared to GG genotype carriers. Higher risk was found in men whom also sustained elevated insulin resistance (IR) compared to these with the GG genotype and lower IR. Moreover, the A allele from *NPAS2* polymorphism was associated with a reduced risk of developing prostate cancer in men with reduced IR when compared to the GG genotype carriers.

Zhu and colleagues also investigated the link between circadian gene with prostate tumors. The case-control study in a Caucasian men population included 1,308 cases and 1,266 control subjects. In this study was genotyped 41 variants in ten genes related with circadian clock (31). At least one polymorphism in nine clock circadian genes was significantly associated with prostate cancer risk. Specifically, it was found the variants rs7950226 in *ARNTL*, rs11133373 in *CLOCK*, rs12315175 in *CRY1*, rs2292912 in *CRY2*, rs1534891 in *CSNK1E*, rs1369481, rs895521, and rs17024926 in *NPAS2*, rs885747 and rs2289591 in *PER1*, rs7602358 in *PER2* and rs1012477 in *PER3*. They observed that the estimate risk for variants rs885747 and rs2289591 in *PER1*, rs1012477 in *PER3*, and rs11133373 in *CLOCK* significantly changed depending on disease aggressiveness.

Lin et al. carried out studies in prostate cancer using two populations, from Seattle and Sweden, respectively) (36). They genotyped 937 polymorphisms corresponding to 156 genes in 1,309 men with prostate cancer in a Seattle cohort. They identified 22 variants associated to prostate cancer-specific mortality (PCSM), and validated them afterward in a Swedish cohort (2,875 patients. In the Swedish cohort, five polymorphisms out of the 22 SNPs identified in the Seattle cohort, were found to be significantly associated with PCSM, with a statistical significance variant in the CRY1 gene (rs10778534). The study also identified another variant in the Seattle cohort, rs228697 in *PER3*, associated with PCSM, which was not further tested in the Swedish cohort due to genotyping drawbacks.

Another study evaluating the association between mortality in prostate cancer and circadian clock-related genes was carried out by Markt et al. (35). Authors tested 96 variants in 12 circadian-related genes using 3 patient cohorts (24, 40, and 105 cases/respectively). It was also analyzed the association with lower levels of melatonin (measured by 6-sulfatoxymelatonin). This study showed no variants significantly associated with overall risk of prostate cancer, however in all cohorts was observed that variation in the *CRY1* gene was associated with mortality in prostate cancer This study of individual cohorts revealed that two polymorphisms from *CRY1*, rs7297614, and rs1921126 were associated to increased mortality in 2 out 3 prostate cancer cohorts, and a similar association was proved for rs12315175 in the *CRY1* gene in a single cohort. Finally, their analysis of the 6-sulfatoxymelatonin levels showed that patients with metabolite levels lower than the median had an increased risk of advanced disease, where polymorphisms in *CSNK1E, NPAS2, PER3*, and *TIMELESS* were associated to changes in these 6-sulfatoxymelatonin production. Future investigations should be designed including a large population compared to the one used in this study, and similar clinicopathological factors as well to ensure statistical power and allow for results comparisons.

Recently, Mocellin et al. performed an analysis using adaptive rank truncated product (ARTP)-based gene and pathway analysis to discern the relevance of the variation in circadian clock genes and cancer susceptibility (4). In this analysis using previously published dataset of prostate cancer (58), they found a highly significant association between genetic variation of circadian pathway and susceptibility to prostate cancer. This result was founded on data regarding 17 SNPs located in seven genes, with the most significant SNP rs142435152 from *ARNTL* gene. Their analysis of subgroups revealed that the risk of suffering aggressive prostate cancer was also highly associated with circadian pathway variation. This finding was based on 28 SNPs located in seven genes, where the most significant gene was *RORA* with the rs17191414 SNP.

## Ovarian Cancer

Ovaries express circadian genes at high levels to regulate hormonal levels during reproductive cycles, and the disruption of this expression is associated to different risk factors for ovarian cancer (e.g., endometriosis).

An association between nightshift work and elevated risk of invasive and borderline ovarian cancers have been described in women aged over 50 years (59). A GWAS and a replication study for epithelial ovarian cancer (EOC) analyzed variants of several circadian genes (ARNTL*, CRY2, CSNK1E, NPAS2, PER3, REV1*, and *TIMELESS*) and two transcription factors (*KLF10* and *SENP3*) (32). The study examined 3,761 EOC patients and 2,722 control subjects. A replication stage was evaluated with ~44,000 subjects with European ancestry. This study indicates that circadian clock genes could act in the development of EOC, particularly *ARNTL* rs10732458, *CSNK1E* rs135750, *SENP3* (rs6608), and *REV1* rs3792152 variants. SNPs in K*LF10* (rs2513928, rs2511703, rs3191333, and rs2513927) were also associated with serious risk of EOC. Interestingly, the most significant association was the rs117104877 variant in *ARNTL*. Ablation of *ARNTL* in mice causes ovary tumors via reduction of p53 expression (60) and dysregulation response to anti-cancer drugs (61). In addition, lower *ARNTL* and *CRY1* expression levels in EOC cells were found after comparison to normal ovarian tissue (62).

Nevertheless, an alternative study has evaluated polymorphism association in nine circadian clock-related genes (*ARNTL, CKIE, CLOCK, CRY1-2, CSNKIE, NPAS2*, and *PER1-3*), but however, it did fail finding any association between genes of the circadian rhythm pathway and ovarian cancer (63).

## Pancreatic and Thyroid Cancer

Pancreatic cancer is a major cause of cancer mortality in western countries population. Several studies have shown disruption of circadian genes expression associated to pancreatic cancer (64–68). However, only one study was able to show an association between circadian genes and pancreatic cancer. It found association between the SNP rs12913421 in *RORA* gene and pancreatic cancer, but the significance disappeared after correcting for multiple comparisons (40). Nevertheless, this study was not specifically designed to evaluate association between polymorphisms in genes of the circadian rhythm pathway and pancreatic cancer. Additional studies of polymorphisms in the circadian pathway are urgently needed, with systematical studies of fine mapping and sufficient sample size.

On the other hand, the disruption of circadian clock genes has been associated to a higher risk of thyroid tumors (69, 70).

Despite this result, no studies have examined the association between polymorphism in circadian genes and thyroid cancer.

## Discussion and Future Perspectives

A better understanding of molecular mechanisms in endocrine-related cancers may facilitate early diagnosis, prognosis and therapies development. Therefore, studies should be improved in several aspects. The reviewed studies about the association of circadian gene polymorphisms to cancer risk have been only conducted in European an East Asian population. Additional studies should be implemented in other ethnic groups to validate epidemiological data.

On the other hand, selection of genetic polymorphisms and experimental and statistical analysis approaches were different among cancer studies and thus, they were not comparable in most cases. These differences might consequently influence the associations found between germline variants and cancer risk.

In future studies, functional implications of the circadian pathway polymorphisms associated to endocrine cancer should be also evaluated. Polymorphisms localized in the 3′UTR could regulate gene transcription. However, the majority of the studied polymorphisms are located in intron regions, thus it is unknown how polymorphisms modulate their functions resulting in a higher or lower cancer risk.

Our review reports that the polymorphisms of some circadian genes are related to cancers of reproductive tissues, where some genes are associated and implicated in three types of tumors (*ARNTL* gene in prostate, breast and ovary), two types of tumors (*CLOCK, CRY1, CRY2, NPAS2*, and *PER2* genes in breast and prostate and *CSNK1E* gene in prostate and ovary) or alternatively they are more specific to one type of cancer (*PER1* and *PER3* genes in prostate and *TIMELESS* gene in breast). Other circadian genes are associated and implicated by several endocrine-related cancers (*RORA* gen in prostate, breast and pancreas).

The current findings suggest that some genes must be involved in the predisposition to cancer development of reproductive tissues, other genes must be specific to a type of cancer, and other genes should affect tissues modulated by endocrine hormones. The effect of these genes is probably showed up at the hormone pathways level, as in the *CLOCK* gene. The activity of the *CLOCK* gen product regulates estrogenic and androgenic hormonal pathways (71, 72). This could be related to the fact that polymorphisms of this gene alter the regulation of these pathways and produce an uncontrolled proliferation of prostate, breast and ovarian tissue cells.

We reckon that a screening of polymorphisms related with the circadian clock could provide valuable information regarding predisposition of suffering a particular type of cancer, thus facilitating its prognosis. When cancer is already present, malignancy intervention strategies could be immediately applied due to earlier detection.

## Concluding Remarks

Compelling evidence supports the notion that loss of circadian homeostasis promotes endocrine cancer development. Genetic component is a key factor that contributes to dysregulation of the circadian clock. Polymorphisms of circadian clock genes are associated to the risk of suffering an endocrine cancer and poor performance to of therapies with anticancer treatments, particularly, these related to reproductive tissues. The findings detailed in this review indicate an exciting research line in order to investigate clock-controlled tumor suppression, also in other organs regulated by circadian rhythms that need high levels of cell proliferation to support their functions, such as thyroid or pancreas.

## Author Contributions

SM-S conceived the project. SM-S, SM, and MM wrote the paper. JL, AC-G, and JJ-L contributed to the revision of literature. All authors have read, corrected, and approved the manuscript.

### Conflict of Interest Statement

The authors declare that the research was conducted in the absence of any commercial or financial relationships that could be construed as a potential conflict of interest.

## References

[1] Fu L Kettner NM. The circadian clock in cancer development and therapy. Prog Mol Biol Transl Sci. (2013) 119:221–82. 10.1016/B978-0-12-396971-2.00009-9 23899600 PMC4103166

[2] Padmanabhan K Billaud M. Desynchronization of circadian clocks in cancer: a metabolic and epigenetic connection. Front Endocrinol (Lausanne) (2017) 8:136. 10.3389/fendo.2017.00136 28674522 PMC5474466

[3] Do MT Yau KW. Intrinsically photosensitive retinal ganglion cells. Physiol Rev. (2010) 90:1547–81. 10.1152/physrev.00013.2010 20959623 PMC4374737

[4] Mocellin S Tropea S Benna C Rossi CR. Circadian pathway genetic variation and cancer risk: evidence from genome-wide association studies. BMC Med. (2018) 16:20. 10.1186/s12916-018-1010-1 29455641 PMC5817863

[5] Gery S Komatsu N Baldjyan L Yu A Koo D Koeffler HP. The circadian gene per1 plays an important role in cell growth and DNA damage control in human cancer cells. Mol Cell (2006) 22:375–82. 10.1016/j.molcel.2006.03.038 16678109

[6] Yang WS Stockwell BR. Inhibition of casein kinase 1-epsilon induces cancer-cell-selective, PERIOD2-dependent growth arrest. Genome Biol. (2008) 9:R92. 10.1186/gb-2008-9-6-r92 18518968 PMC2481424

[7] Jenwitheesuk A Nopparat C Mukda S Wongchitrat P Govitrapong P. Melatonin regulates aging and neurodegeneration through energy metabolism, epigenetics, autophagy and circadian rhythm pathways. Int J Mol Sci. (2014) 15:16848–84. 10.3390/ijms150916848 25247581 PMC4200827

[8] Angelousi A Kassi E Nasiri-Ansari N Weickert MO Randeva H Kaltsas G. Clock genes alterations and endocrine disorders. Eur J Clin Invest. (2018) 48:e12927. 10.1111/eci.12927 29577261

[9] Valenzuela FJ Vera J Venegas C Munoz S Oyarce S Munoz K. Evidences of polymorphism associated with circadian system and risk of pathologies: a review of the literature. Int J Endocrinol. (2016) 2016:2746909. 10.1155/2016/2746909 27313610 PMC4893437

[10] Schibler U Ripperger J Brown SA. Peripheral circadian oscillators in mammals: time and food. J Biol Rhythms (2003) 18:250–60. 10.1177/0748730403018003007 12828282

[11] Liu F Chang HC. Physiological links of circadian clock and biological clock of aging. Protein Cell (2017) 8:477–88. 10.1007/s13238-016-0366-2 28108951 PMC5498335

[12] Dibner C Schibler U Albrecht U. The mammalian circadian timing system: organization and coordination of central and peripheral clocks. Annu Rev Physiol. (2010) 72:517–49. 10.1146/annurev-physiol-021909-135821 20148687

[13] Benna C Helfrich-Forster C Rajendran S Monticelli H Pilati P Nitti D. Genetic variation of clock genes and cancer risk: a field synopsis and meta-analysis. Oncotarget (2017) 8:23978–95. 10.18632/oncotarget.15074 28177907 PMC5410358

[14] Ikeda M Nomura M. cDNA cloning and tissue-specific expression of a novel basic helix-loop-helix/PAS protein (BMAL1) and identification of alternatively spliced variants with alternative translation initiation site usage. Biochem Biophys Res Commun. (1997) 233:258–64. 10.1006/bbrc.1997.6371 9144434

[15] Yu W Ikeda M Abe H Honma S Ebisawa T Yamauchi T. Characterization of three splice variants and genomic organization of the mouse BMAL1 gene. Biochem Biophys Res Commun. (1999) 260:760–7. 10.1006/bbrc.1999.0970 10403839

[16] King DP Zhao Y Sangoram AM Wilsbacher LD Tanaka M Antoch MP. Positional cloning of the mouse circadian clock gene. Cell (1997) 89:641–53. 10.1016/S0092-8674(00)80245-7 9160755 PMC3815553

[17] Hsu DS Zhao X Zhao S Kazantsev A Wang RP Todo T. Putative human blue-light photoreceptors hCRY1 and hCRY2 are flavoproteins. Biochemistry (1996) 35:13871–7. 10.1021/bi962209o 8909283

[18] Shearman LP Zylka MJ Weaver DR Kolakowski LF Jr Reppert SM. Two period homologs: circadian expression and photic regulation in the suprachiasmatic nuclei. Neuron (1997) 19:1261–9. 10.1016/S0896-6273(00)80417-1 9427249

[19] Tei H Okamura H Shigeyoshi Y Fukuhara C Ozawa R Hirose M. Circadian oscillation of a mammalian homologue of the Drosophila period gene. Nature (1997) 389:512–6. 10.1038/39086 9333243

[20] Kim P Oster H Lehnert H Schmid SM Salamat N Barclay JL. Coupling the circadian clock to homeostasis: the role of Period in timing physiology. Endocr Rev. (2018) 40:66–95. 10.1210/er.2018-00049 30169559

[21] Zheng X Sowcik M Chen D Sehgal A. Casein kinase 1 promotes synchrony of the circadian clock network. Mol Cell Biol. (2014) 34:2682–94. 10.1128/MCB.01571-13 24820422 PMC4097653

[22] Reick M Garcia JA Dudley C McKnight SL. NPAS2: an analog of clock operative in the mammalian forebrain. Science (2001) 293:506–9. 10.1126/science.1060699 11441147

[23] Landgraf D Wang LL Diemer T Welsh DK. NPAS2 Compensates for loss of CLOCK in peripheral circadian oscillators. PLoS Genet. (2016) 12:e1005882. 10.1371/journal.pgen.1005882 26895328 PMC4760943

[24] Preitner N Damiola F Lopez-Molina L Zakany J Duboule D Albrecht U. The orphan nuclear receptor REV-ERBalpha controls circadian transcription within the positive limb of the mammalian circadian oscillator. Cell (2002) 110:251–60. 10.1016/S0092-8674(02)00825-5 12150932

[25] Kim YH Marhon SA Zhang Y Steger DJ Won KJ Lazar MA. Rev-erbalpha dynamically modulates chromatin looping to control circadian gene transcription. Science (2018) 359:1274–7. 10.1126/science.aao6891 29439026 PMC5995144

[26] Cho H Zhao X Hatori M Yu RT Barish GD Lam MT. Regulation of circadian behaviour and metabolism by REV-ERB-alpha and REV-ERB-beta. Nature (2012) 485:123–7. 10.1038/nature11048 22460952 PMC3367514

[27] Sato TK Panda S Miraglia LJ Reyes TM Rudic RD McNamara P. A functional genomics strategy reveals Rora as a component of the mammalian circadian clock. Neuron (2004) 43:527–37. 10.1016/j.neuron.2004.07.018 15312651

[28] Gotter AL Manganaro T Weaver DR Kolakowski LF Jr Possidente B Sriram S. A time-less function for mouse timeless. Nat Neurosci. (2000) 3:755–6. 10.1038/77653 10903565

[29] Mazzoccoli G Laukkanen MO Vinciguerra M Colangelo T Colantuoni V. A timeless link between circadian patterns and disease. Trends Mol Med. (2016) 22:68–81. 10.1016/j.molmed.2015.11.007 26691298

[30] Rabstein S Harth V Justenhoven C Pesch B Plottner S Heinze E. Polymorphisms in circadian genes, night work and breast cancer: results from the GENICA study. Chronobiol Int. (2014) 31:1115–22. 10.3109/07420528.2014.957301 25229211

[31] Zhu Y Stevens RG Hoffman AE Fitzgerald LM Kwon EM Ostrander EA. Testing the circadian gene hypothesis in prostate cancer: a population-based case-control study. Cancer Res. (2009) 69:9315–22. 10.1158/0008-5472.CAN-09-0648 19934327 PMC2955869

[32] Jim HS Lin HY Tyrer JP Lawrenson K Dennis J Chornokur G. Common genetic variation in circadian rhythm genes and risk of epithelial ovarian cancer (EOC). J Genet Genome Res. (2015) 2:017. 10.23937/2378-3648/1410017 26807442 PMC4722961

[33] Dai H Zhang L Cao M Song F Zheng H Zhu X. The role of polymorphisms in circadian pathway genes in breast tumorigenesis. Breast Cancer Res Treat. (2011) 127:531–40. 10.1007/s10549-010-1231-2 20978934

[34] Zhu Y Brown HN Zhang Y Stevens RG Zheng T. Period3 structural variation: a circadian biomarker associated with breast cancer in young women. Cancer Epidemiol Biomarkers Prev. (2005) 14:268–70. Available online at: http://cebp.aacrjournals.org/content/14/1/268.long 15668506

[35] Markt SC Valdimarsdottir UA Shui IM Sigurdardottir LG Rider JR Tamimi RM. Circadian clock genes and risk of fatal prostate cancer. Cancer Causes Control. (2015) 26:25–33. 10.1007/s10552-014-0478-z 25388799 PMC4282953

[36] Lin DW FitzGerald LM Fu R Kwon EM Zheng SL Kolb S. Genetic variants in the LEPR, CRY1, RNASEL, IL4, and ARVCF genes are prognostic markers of prostate cancer-specific mortality. Cancer Epidemiol Biomarkers Prev. (2011) 20:1928–36. 10.1158/1055-9965.EPI-11-0236 21846818 PMC3169727

[37] Chu LW Zhu Y Yu K Zheng T Yu H Zhang Y. Variants in circadian genes and prostate cancer risk: a population-based study in China. Prostate Cancer Prostatic Dis. (2008) 11:342–8. 10.1038/sj.pcan.4501024 17984998

[38] Monsees GM Kraft P Hankinson SE Hunter DJ Schernhammer ES. Circadian genes and breast cancer susceptibility in rotating shift workers. Int J Cancer (2012) 131:2547–52. 10.1002/ijc.27564 22473669 PMC3408553

[39] Truong T Liquet B Menegaux F Plancoulaine S Laurent-Puig P Mulot C. Breast cancer risk, nightwork, and circadian clock gene polymorphisms. Endocr Relat Cancer (2014) 21:629–38. 10.1530/ERC-14-0121 24919398

[40] Cotterchio M Lowcock E Bider-Canfield Z Lemire M Greenwood C Gallinger S. Association between variants in atopy-related immunologic candidate genes and pancreatic cancer risk. PLoS ONE (2015) 10:e0125273. 10.1371/journal.pone.0125273 25945796 PMC4422524

[41] Fu A Leaderer D Zheng T Hoffman AE Stevens RG Zhu Y. Genetic and epigenetic associations of circadian gene TIMELESS and breast cancer risk. Mol Carcinog. (2012) 51:923–9. 10.1002/mc.20862 22006848

[42] Ijaz S Verbeek J Seidler A Lindbohm ML Ojajarvi A Orsini N Night-shift work and breast cancer–a systematic review and meta-analysis. Scand J Work Environ Health (2013) 39:431–47. 10.5271/sjweh.3371 23804277

[43] Blakeman V Williams JL Meng QJ Streuli CH. Circadian clocks and breast cancer. Breast Cancer Res. (2016) 18:89. 10.1186/s13058-016-0743-z 27590298 PMC5010688

[44] Wegrzyn LR Tamimi RM Rosner BA Brown SB Stevens RG Eliassen AH. Rotating night-shift work and the risk of breast cancer in the Nurses' health studies. Am J Epidemiol. (2017) 186:532–40. 10.1093/aje/kwx140 28541391 PMC5856106

[45] Johns LE Jones ME Schoemaker MJ McFadden E Ashworth A Swerdlow AJ Domestic light at night and breast cancer risk: a prospective analysis of 105,000 UK women in the Generations Study. Br J Cancer (2018) 118:600–6. 10.1038/bjc.2017.359 29360812 PMC5830585

[46] Lesicka M Jablonska E Wieczorek E Seroczynska B Siekierzycka A Skokowski J. Altered circadian genes expression in breast cancer tissue according to the clinical characteristics. PLoS ONE (2018) 13:e0199622. 10.1371/journal.pone.0199622 29958276 PMC6025856

[47] Lee C Weaver DR Reppert SM. Direct association between mouse PERIOD and CKIepsilon is critical for a functioning circadian clock. Mol Cell Biol. (2004) 24:584–94. 10.1128/MCB.24.2.584-594.2004 14701732 PMC343819

[48] Yang X Wood PA Oh EY Du-Quiton J Ansell CM Hrushesky WJ. Down regulation of circadian clock gene Period 2 accelerates breast cancer growth by altering its daily growth rhythm. Breast Cancer Res Treat. (2009) 117:423–31. 10.1007/s10549-008-0133-z 18651214

[49] Rossetti S Corlazzoli F Gregorski A Azmi NH Sacchi N. Identification of an estrogen-regulated circadian mechanism necessary for breast acinar morphogenesis. Cell Cycle (2012) 11:3691–700. 10.4161/cc.21946 22935699 PMC3478319

[50] Kochan DZ Kovalchuk O. Circadian disruption and breast cancer: an epigenetic link? Oncotarget (2015) 6:16866–82. 10.18632/oncotarget.4343 26220712 PMC4627279

[51] Li J Humphreys K Heikkinen T Aittomaki K Blomqvist C Pharoah PD. A combined analysis of genome-wide association studies in breast cancer. Breast Cancer Res Treat. (2011) 126:717–27. 10.1007/s10549-010-1172-9 20872241

[52] Jemal A Siegel R Ward E Hao Y Xu J Thun MJ. Cancer statistics, 2009. CA Cancer J Clin. (2009) 59:225–49. 10.3322/caac.20006 19474385

[53] Plymate SR Tenover JS Bremner WJ. Circadian variation in testosterone, sex hormone-binding globulin, and calculated non-sex hormone-binding globulin bound testosterone in healthy young and elderly men. J Androl. (1989) 10:366–71. 10.1002/j.1939-4640.1989.tb00120.x 2592266

[54] Feldman BJ Feldman D. The development of androgen-independent prostate cancer. Nat Rev Cancer (2001) 1:34–45. 10.1038/35094009 11900250

[55] Fu L Lee CC. The circadian clock: pacemaker and tumour suppressor. Nat Rev Cancer (2003) 3:350–61. 10.1038/nrc1072 12724733

[56] Cao Q Gery S Dashti A Yin D Zhou Y Gu J. A role for the clock gene per1 in prostate cancer. Cancer Res. (2009) 69:7619–25. 10.1158/0008-5472.CAN-08-4199 19752089 PMC2756309

[57] Kiss Z Ghosh PM. WOMEN IN CANCER THEMATIC REVIEW: Circadian rhythmicity and the influence of ‘clock’ genes on prostate cancer. Endocr Relat Cancer (2016) 23:T123–T134. 10.1530/ERC-16-0366 27660402 PMC5148656

[58] Al Olama AA Kote-Jarai Z Berndt SI Conti DV Schumacher F Han Y. A meta-analysis of 87,040 individuals identifies 23 new susceptibility loci for prostate cancer. Nat Genet. (2014) 46:1103–9. 10.1038/ng.3094 25217961 PMC4383163

[59] Bhatti P Cushing-Haugen KL Wicklund KG Doherty JA Rossing MA. Nightshift work and risk of ovarian cancer. Occup Environ Med. (2013) 70:231–7. 10.1136/oemed-2012-101146 23343856 PMC3777774

[60] Lee S Donehower LA Herron AJ Moore DD Fu L. Disrupting circadian homeostasis of sympathetic signaling promotes tumor development in mice. PLoS ONE (2010) 5:e10995. 10.1371/journal.pone.0010995 20539819 PMC2881876

[61] Zeng ZL Wu MW Sun J Sun YL Cai YC Huang YJ. Effects of the biological clock gene Bmal1 on tumour growth and anti-cancer drug activity. J Biochem. (2010) 148:319–26. 10.1093/jb/mvq069 20576619

[62] Tokunaga H Takebayashi Y Utsunomiya H Akahira J Higashimoto M Mashiko M. Clinicopathological significance of circadian rhythm-related gene expression levels in patients with epithelial ovarian cancer. Acta Obstet Gynecol Scand. (2008) 87:1060–70. 10.1080/00016340802348286 18720043

[63] Gu F Zhang H Hyland PL Berndt S Gapstur SM Wheeler W. Inherited variation in circadian rhythm genes and risks of prostate cancer and three other cancer sites in combined cancer consortia. Int J Cancer (2017) 141:1794–802. 10.1002/ijc.30883 28699174 PMC5907928

[64] Oda A Katayose Y Yabuuchi S Yamamoto K Mizuma M Shirasou S. Clock gene mouse period2 overexpression inhibits growth of human pancreatic cancer cells and has synergistic effect with cisplatin. Anticancer Res. (2009) 29:1201–9. Available online at: http://ar.iiarjournals.org/content/29/4/1201.long 19414365

[65] Wu Y Sato F Yamada T Bhawal UK Kawamoto T Fujimoto K. The BHLH transcription factor DEC1 plays an important role in the epithelial-mesenchymal transition of pancreatic cancer. Int J Oncol. (2012) 41:1337–46. 10.3892/ijo.2012.1559 22825629

[66] Relles D Sendecki J Chipitsyna G Hyslop T Yeo CJ Arafat HA. Circadian gene expression and clinicopathologic correlates in pancreatic cancer. J Gastrointest Surg. (2013) 17:443–50. 10.1007/s11605-012-2112-2 23254314

[67] Tavano F Pazienza V Fontana A Burbaci FP Panebianco C Saracino C. SIRT1 and circadian gene expression in pancreatic ductal adenocarcinoma: effect of starvation. Chronobiol Int. (2015) 32:497–512. 10.3109/07420528.2014.1003351 25798752

[68] Jiang W Zhao S Jiang X Zhang E Hu G Hu B. The circadian clock gene Bmal1 acts as a potential anti-oncogene in pancreatic cancer by activating the p53 tumor suppressor pathway. Cancer Lett. (2016) 371:314–25. 10.1016/j.canlet.2015.12.002 26683776

[69] Mannic T Meyer P Triponez F Pusztaszeri M Le Martelot G Mariani O. Circadian clock characteristics are altered in human thyroid malignant nodules. J Clin Endocrinol Metab. (2013) 98:4446–56. 10.1210/jc.2013-2568 23979949

[70] Wilson C. Cancer: Thyroid circadian clock–altered in cancer? Nat Rev Endocrinol. (2013) 9:628. 10.1038/nrendo.2013.182 24042329

[71] Zhu Y Zheng T Stevens RG Zhang Y Boyle P. Does “clock” matter in prostate cancer? Cancer Epidemiol Biomarkers Prev. (2006) 15:3–5. 10.1158/1055-9965.EPI-05-0631 16434577 PMC2366206

[72] Li S Wang M Ao X Chang AK Yang C Zhao F. CLOCK is a substrate of SUMO and sumoylation of CLOCK upregulates the transcriptional activity of estrogen receptor-alpha. Oncogene (2013) 32:4883–91. 10.1038/onc.2012.518 23160374

